# From the niche to malignant hematopoiesis and back: reciprocal interactions between leukemia and the bone marrow microenvironment

**DOI:** 10.1002/jbm4.10516

**Published:** 2021-06-03

**Authors:** Celia A. Soto, Cristina Lo Celso, Louise E. Purton, Benjamin J. Frisch

**Affiliations:** ^1^ Department of Pathology University of Rochester Medical Center Rochester New York USA; ^2^ Department of Life Sciences Imperial College London London UK; ^3^ Sir Francis Crick Institute London UK; ^4^ St Vincent's Institute of Medical Research Fitzroy Victoria Australia; ^5^ Department of Medicine at St. Vincent's Hospital The University of Melbourne Melbourne Victoria Australia; ^6^ Wilmot Cancer Institute University of Rochester School of Medicine and Dentistry Rochester New York USA; ^7^ Center for Musculoskeletal Research University of Rochester School of Medicine and Dentistry Rochester New York USA

**Keywords:** AGING, BONE, CANCER, CHEMOTHERAPY, HEMATOPOIESIS, HEMATOPOIETIC STEM CELL (HSC) NICHE, INFLAMMATION, LEUKEMIA, MICROENVIRONMENT

## Abstract

The bone marrow microenvironment (BMME) regulates hematopoiesis through a complex network of cellular and molecular components. Hematologic malignancies reside within, and extensively interact with, the same BMME. These interactions consequently alter both malignant and benign hematopoiesis in multiple ways, and can encompass initiation of malignancy, support of malignant progression, resistance to chemotherapy, and loss of normal hematopoiesis. Herein, we will review supporting studies for interactions of the BMME with hematologic malignancies and discuss challenges still facing this exciting field of research. © 2021 The Authors. *JBMR Plus* published by Wiley Periodicals LLC on behalf of American Society for Bone and Mineral Research.

## Introduction

### The bone marrow microenvironment is at the core of normal blood cell production

The bone marrow microenvironment (BMME) encompasses the cellular and extracellular components that cooperate to regulate maintenance of the skeleton and hematopoiesis. Postnatally, all blood cells arise from multipotent hematopoietic stem cells (HSCs)^(^ ^1^ ^)^ with the vast majority of these cells located in the bone marrow (BM). The HSC “niche” is typically thought of as a site in the bone where HSCs reside when not circulating, and where they receive signals from a complex network of cells to remain quiescent, self‐renew, or differentiate into one of multiple hematopoietic lineages.^(^ ^2^ ^)^ Today, we know that several HSC niches exist in different areas within the BM, including the arteriolar and sinusoidal niches, which are composed of multiple and often overlapping stromal and hematopoietic cell types. However, different variations of the niches are more specialized in the production and maturation of the various types of committed hematopoietic progenitors, such as common myeloid and common lymphoid progenitors.

The described cellular and molecular components of the HSC niche^(^ ^3^ ^)^ are continuously expanding, and today include both endosteal and endothelial components such as: mesenchymal stromal cells (MSCs),^(^ ^4^, ^5^, ^6^ ^)^ arteriolar^(^ ^7^ ^)^ and sinusoidal^(^ ^8^, ^9^ ^)^ endothelial cells (ECs), osteoblasts (OBs),^(^ ^10^, ^11^, ^12^, ^13^ ^)^ spindle‐shaped N‐cadherin^+^CD45^−^ osteoblastic cells (SNO cells),^(^ ^12^ ^)^ macrophages,^(^ ^14^ ^)^ megakaryocytes,^(^ ^15^ ^)^ T cells,^(^ ^16^ ^)^ the sympathetic nervous system (SNS) through β‐adrenergic signaling,^(^ ^17^, ^18^ ^)^ perivascular stromal cells^(^ ^19^ ^)^ including LepR^+^ skeletal stem cells,^(^ ^20^ ^)^ chemokine (C‐X‐C motif) ligand 12 (CXCL12)‐abundant reticular (CAR) cells,^(^ ^21^ ^)^ skeletal lineage cells,^(^ ^22^ ^)^ Schwann cells,^(^ ^23^ ^)^ and adipocytes.^(^ ^24^, ^25^ ^)^ These niche cells produce numerous regulatory factors that are vital for HSC quiescence and regeneration, and control of differentiation, including various cell‐surface proteins, extracellular cytokines, and inflammatory molecules, such as CXCL12,^(^ ^5^, ^21^ ^)^ angiopoietin‐1 (Ang‐1),^(^ ^26^ ^)^ thrombopoietin (TPO),^(^ ^27^ ^)^ stem cell factor (SCF),^(^ ^28^ ^)^ C‐C motif chemokine ligand 3 (CCL3),^(^ ^29^, ^30^ ^)^ and angiogenin (ANG),^(^ ^31^ ^)^ as well as variable oxygen tension and reactive oxygen species (ROS).^(^ ^32^, ^33^, ^34^ ^)^

### Hematological malignancies have reciprocal interactions with BM cell signals

The BMME plays a critical role in the development of malignant hematopoiesis and leukemic progression in various mouse models. Alterations to normal microenvironment signaling can initiate hematologic malignancies, in conjunction with leukemic BM then contributing signals that alter hematopoietic regulation. Interactions with the BMME can also allow leukemic cells to escape chemotherapy treatment.^(^ ^35^ ^)^ Therefore, in addition to classically targeting cell‐intrinsic mechanisms through chemotherapy, targeting the leukemic cell‐extrinsic microenvironment may be therapeutically beneficial.^(^ ^36^, ^37^, ^38^ ^)^ Despite the distinct biological differences among subtypes of leukemia, they all reside in the BM and involve BMME alterations and interactions, especially with cells that compose the HSC niche. For these reasons, investigation of microenvironmental dysfunction from one type of leukemia may be broadly applicable across multiple hematologic malignancies. In this review we discuss the BMME as it applies across various types of leukemias.

## Microenvironment Support for the Initiation of Malignant Hematopoiesis

**Fig. 1:**
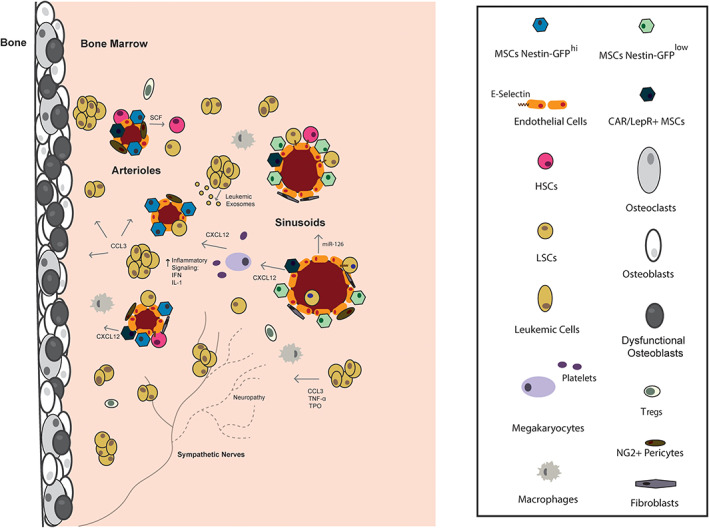
Malignant cells interact with the BMME at the HSC niche: HSC niches in the BM are endosteal and perivascular. The bone surface is covered with osteoblastic lineage cells, including OBs and osteoclasts. Arterioles (cross‐sectional view) and sympathetic nerves are the site of the arteriolar niche, spatially located with Nestin‐GFP^hi^ MSCs and NG2^+^ pericytes, which secrete factors for HSC maintenance such as SCF. Closer to the center of the BM are sinusoids (cross‐sectional view), where a sinusoidal HSC niche is comprised of CAR/LepR^+^ cells, Nestin‐GFP^‐lo^ MSCs, perivascular fibroblasts/NG2+ pericytes, and megakaryocytes. HSCs, LSCs, and progeny extravasate through the sinusoids to circulation for hematopoiesis. HSCs adhere at the niche, and this maintains quiescent reserve HSCs and provides signals for self‐renewal. During malignancy: (i) LSCs can occupy the niche, by adhering to E‐selectin on ECs, where stromal cells and fibroblasts confer signals that contribute to chemotherapy resistance, (ii) ECs, fibroblasts, osteoblast lineage cells, and megakaryocytes secrete excess CXCL12 to the BMME, (iii) ECs secrete miR‐126, which promotes LSC self‐renewal, (iv) leukemia cells secrete exosomes that suppress stromal cell function and osteoblast development, (v) loss of sympathetic nerves is linked to loss of support for Nestin^+^ MSCs, (vi) immune cells such as Tregs and leukemia‐supportive macrophages provide immunosuppressive signals, (vii) leukemic blasts accumulate in the BMME around the HSC niches, where they secrete excess CCL3, TNF, and TPO, (viii) there is an increase in inflammatory signaling, such as IFN‐γ and IL‐1 and, (ix) CCL3 leads to the formation of defective osteoblasts. Abbreviations: BM, bone marrow; BMME, bone marrow microenvironment; CAR/LepR^+^, CXCL12‐abundant reticular cells and leptin receptor+ cells; CCL3, C‐C motif chemokine ligand 3; CXCL12, chemokine (C‐X‐C motif) ligand 12; EC, endothelial cell; GFP, green fluorescent protein; HSC, hematopoietic stem cell; IFN‐γ, interferon γ; IL‐1, interleukin 1; LSC, leukemia stem cell; miR‐126, microRNA‐126; MSC, mesenchymal stromal cell; NG2, Neuron‐Glial Antigen 2; OB, osteoblast; SCF, stem cell factor; TNF, tumor necrosis factor; TPO, thrombopoietin; Treg, T regulatory cell.

It has become well‐established that a disruption of homeostasis in the BMME can initiate leukemia in murine models. It was originally thought that leukemia develops solely from cell‐intrinsic mechanisms, such as somatic mutations, leading to cancer. Although this is an important mechanism of disease initiation, the other cellular components of the BMME are also susceptible to dysfunction caused by aging and chronic inflammation, and can play an important role in the induction of malignant hematopoiesis and leukemias; therefore, a knowledge of HSC‐extrinsic mechanisms may be important for understanding leukemia pathogenesis. In murine models, several specific genetic alterations to the microenvironment have been shown to lead to myeloproliferative disease, even when healthy BM HSCs (from wild‐type mice) were transplanted into the diseased BM. Support for leukemic development from the BMME thus far has largely been focused on ECs and the osteolineage cells, including MSCs and OBs. Last, changes that occur in aged BM contribute to the development of leukemias. Although there are barriers to studying leukemic development in humans, the use of mouse models has given insight to the importance of BM microenvironmental signaling to the development of hematological malignancies (Figure 1).

### Aberrant signaling in the BMME can result in myeloproliferation

Retinoic acid receptors (RARs) are nuclear hormone receptors implicated in the generation of HSCs and normal hematopoiesis. The deletion of the retinoic acid receptor‐γ (RAR‐γ) in mice, using a homozygous mutant null mouse for the *Rarg* gene, led to the development of a myeloproliferative‐like syndrome (MPS‐like).^(^ ^39^ ^)^ When BM from wild‐type mice was transplanted into *Rarg* ^−/−^ mice, the MPS‐like phenotype occurred, featuring overproduction of granulocytes and granulocyte/macrophage progenitors in the BM, spleen, and peripheral blood. In contrast, when *Rarg* ^−/−^ BM was transplanted into wild‐type recipients, hematopoiesis was restored to normal. Therefore, the presence of signaling from this receptor in the BMME will dictate whether MPS‐like symptoms will form, even when the HSCs are normal and healthy.

Under stress conditions, cell‐cycle proteins such as the retinoblastoma protein (Rb)‐family proteins influence HSCs to leave quiescence, and cycle for production of the myeloid or lymphoid lineage cells.^(^ ^40^ ^)^ When the *Rb* gene was deleted in the BMME using an interferon (IFN)‐inducible *Mx1*‐Cre^+^ *pRb* ^Δ/Δ^ mouse model, a myeloproliferative phenotype was observed; this was accompanied by profound mobilization of HSCs from the BM.^(^ ^41^ ^)^ In contrast, deletion of *Rb* in HSCs alone resulted in only subtle changes in hematopoiesis.^(^ ^42^ ^)^ The myeloproliferation observed in *Mx1*‐Cre^+^ *pRb* ^Δ/Δ^ mice may be attributed to dysfunctional cellular interactions in the BMME, from altered crosstalk between the niche cells and *Rb*‐deficient myeloid‐derived cells.

Notch signaling is necessary for hematopoiesis and is associated with β‐catenin signaling in BM cells local to the HSC niche.^(^ ^11^, ^43^, ^44^ ^)^ When conditional knockout mice were generated to have inactive Mind bomb‐1 (Mib1), a protein involved in the endocytosis of the Notch ligand, by use of a cyclic recombinase–locus of x‐over (Cre‐Lox) system under the control of the promoters *MMTV* and *Mx1*, defective Notch signaling occurred and resulted in MPS‐like disease.^(^ ^45^ ^)^ Additionally, MPS‐like disease still formed upon transplantation of wild‐type BM into the Mib1‐null mice, providing evidence for the contribution of Notch signaling in the BMME to the disease pathogenesis. Together, these studies have demonstrated that a single genetic alteration to BM signaling can provide a cellular environment with sufficient support to develop myeloproliferative diseases, without an oncogenic mutation in the HSCs.

### Leukemogenesis can arise from alterations to various BM cells

Mutations in osteoprogenitors alone have led to leukemic conditions in mouse models. The deletion of the ribonuclease III enzyme *Dicer1* from mouse osteoprogenitors, through the use of Cre recombinase driven by the osterix promoter (*Osx*‐Cre), led to myelodysplasia (MDS) (reduced blood cell counts with abnormalities detected in at least one blood cell lineage) and phenotypes typical of acute myeloid leukemia (AML), such as the accumulation of immature blood cells.^(^ ^46^ ^)^ On the other hand, this was not observed when *Dicer1* was deleted from mature OBs, by using a Cre recombinase driven by the osteocalcin promoter. Moreover, due to the *Dicer1* deletion, lower levels of Shwachman‐Bodian Diamond syndrome (SBDS) ribosome maturation factor were expressed in the osteoprogenitors, and when the *Sbds* gene was deleted from osteoprogenitors through *Osx*‐Cre in a subsequent experiment, it again resulted in a myelodysplasia, confirming the role of this signaling pathway in the disease pathogenesis.

In murine osteoprogenitors and MSCs, a mutation of the gene *Ptpn11*, causing activation of the protein tyrosine phosphatase SHP2, can lead to myeloproliferative neoplasm and subsequent leukemogenesis, and this effect was not seen if SHP2 was similarly activated in mature osteolineage cells.^(^ ^47^ ^)^ As a result of this mutation in the progenitors, high levels of the protein CCL3, also called macrophage inflammatory protein‐1 alpha (MIP‐1α), are secreted to the BMME, altering the HSC niche by recruiting monocytes and elevating the levels of proinflammatory cytokines in the local microenvironment. Another study confirmed that this inflammatory signaling pathway is upregulated across a variety of human pre‐leukemic syndromes, and it predicted leukemic progression and prognosis in MDS.^(^ ^48^ ^)^ These studies demonstrate that osteoprogenitors regulate normal hematopoiesis and can lead to a neoplastic pre‐leukemic disease when mutated or dysfunctional.

Mature OBs with mutations have been found to be capable of initiating leukemia. Expression of a constitutively active β‐catenin in mouse OBs resulted in overexpression of the Notch ligand Jagged1 (JAG1), and subsequent development of AML.^(^ ^43^ ^)^ Furthermore, inhibition of the Notch pathway in an AML model lessened leukemic development. Additionally, the Jag1‐Notch signaling pathway was further confirmed to be upregulated in the OBs of over one‐third of human AML patients. Therefore, JAG1 is necessary for normal hematopoiesis, dysregulated Notch signaling in the BMME may contribute to the development of leukemias, and targeting this signaling pathway presents a therapeutic technique to slow disease progression in humans.

ECs are a major component of all HSC niches, lining the vessel walls of the arterioles and sinusoids, and participating in the hematopoietic signaling network. Deletion of the gene for the receptor subunit glycoprotein 130 (gp130), a component of the receptors for interleukin 6 (IL‐6) family of cytokines, by use of a Cre/loxP‐mediated recombination driven by the *Tie2* promoter (*Tie2*‐Cre), resulted in BM dysfunction and myeloproliferation.^(^ ^49^ ^)^ These hematopoietic defects were observed when gp130 remained expressed by other stromal cell types, supporting a role unique to ECs in homeostatic blood cell production. To note, in this model, gp130 was also deleted in the hematopoietic cells, as they too are targeted by *Tie2*‐Cre; however, when wild‐type BM was transplanted into gp130*‐*deficient BMME the hematopoietic defects were replicated.^(^ ^49^ ^)^ This confirmed the importance of gp130 on BM ECs alone as an HSC‐extrinsic regulator in the BMME that is required for normal hematopoiesis, and that a myeloproliferative disorder can result from EC dysfunction or alteration.

### Aged BMME as a risk factor for leukemic initiation

Hematological malignancies commonly develop and are diagnosed in older individuals.^(^ ^50^, ^51^, ^52^, ^53^ ^)^ This is attributed in part to aged HSCs, which become genetically and epigenetically altered due the natural accumulation of spontaneous somatic mutations over time, leading to the development of clonal hematopoiesis from mutations that confer a dominant proliferative phenotype.^(^ ^54^ ^)^ However, the following cell‐extrinsic mechanisms within the aging BMME also contribute to malignancy initiation.

Chronic inflammation increases with age, sometimes referred to as the phenomenon of “inflammaging”. This results in a higher percentage of white blood cells present in the BM, secreting a higher concentration of inflammatory cytokines.^(^ ^55^ ^)^ For example, extended exposure to high levels of proinflammatory signaling by ILs, that occurs during “emergency” hematopoiesis, affects HSC differentiation during chronic inflammation,^(^ ^56^ ^)^ although it is still unknown whether this is sensed directly by the HSCs or conferred through alterations in BMME signaling.

MSCs decrease in number and functionality with aging, as they become senescent,^(^ ^57^ ^)^ including a reduced capacity to differentiate to osteogenic cells; instead, they produce an above‐normal ratio of adipocytes in the BMME.^(^ ^58^ ^)^ Increased adipose tissue and ectopic deposition of lipids can remodel the niche architecture, and increase inflammation in the BM, thereby reducing niche function as has been observed by a lessened capacity for hematopoietic reconstitution.^(^ ^24^, ^59^ ^)^

There is a well‐described tendency toward the production of myeloid cells by the aged hematopoietic system, referred to as “myeloid bias”. Vascular remodeling occurs with aging in the BM, and this manifests as a decreased number of arterioles with fewer Nestin^+^ MSCs to support the HSC niche.^(^ ^60^ ^)^ In addition, the endosteal arterioles contract while the Notch ligand Jag2 increases at the sinusoids; together, this directs the location of HSCs towards the sinusoidal niche.^(^ ^61^ ^)^ The arteriolar niche is preferential to production of lymphoid progenitors from HSCs,^(^ ^62^ ^)^ so this loss of arteriolar niche signaling contributes to the shift toward myelopoiesis displayed in aging. Additionally, signaling from the inflammatory cytokine “regulated on activation, normal T cell expressed and secreted” (Rantes) has been found to promote myeloid bias in aged BM, and when aged HSCs are placed in a young BMME the overproduction of myeloid cells is reduced.^(^ ^63^ ^)^

Aged macrophages play a role in the bias toward the production of megakaryocytes by aged HSCs through increased IL‐1β production in the BM.^(^ ^64^ ^)^ The skewed myelopoiesis, and dysfunction of aged BM macrophages, also results in the accumulation of senescent neutrophils in the BM, further driving HSC niche dysfunction in aging.^(^ ^64^ ^)^ Aged neutrophils that infiltrate the HSC niche are dysfunctional in their homeostatic role in the clearance of dying cells in the BM, and this distorts signaling that controls the circadian‐rhythm related egress of hematopoietic progenitors to and from circulation.^(^ ^65^ ^)^

### Barriers to the study of the BMME

There are inherent difficulties when using murine models for study of the leukemic initiation and progression in the BMME. Cell specificity of the Cre strains is one such caveat to be aware of when designing studies.^(^ ^66^ ^)^ Of note, it is unclear why hematologic malignancy induced in mouse models by alterations to the microenvironment has largely been of myeloid rather than lymphoid origin. Perhaps myeloid differentiation is more sensitive to signals from the BMME, and therefore to microenvironmental dysfunction. It is also possible that this occurs because many of the deregulated BMME factors have included inflammatory cytokines, which favor myeloid cell expansion.^(^ ^67^ ^)^ Alternatively, or additionally, this may be due to the specific inbred strains of mice used, typically C57BL/6; however, this strain has been more commonly predisposed to the natural development of lymphoid malignancies.^(^ ^68^ ^)^ Ultimately, the rationale behind these predispositions has not been elucidated; spontaneous and extrinsically induced malignancies differ, and further research into the mechanisms of extrinsic and intrinsic leukemic initiation is still needed, as well as confirming these findings on additional strains of mice.

Studying the contribution of the BMME to disease development in humans has been hindered for multiple reasons; for example, the leukemic cells express many of the same markers that would be used to identify mature blood cells, and performing BM biopsies removes the cells from the architecture of the niche and thereby away from the signals that drive dysfunction. Because of such barriers to studying the role of the BMME directly in leukemia patients, the majority of the research performed has made use of murine models of disease. Therefore, confirmation of these findings using human in vitro disease models, and in human clinical studies, is paramount for translational applications. Nevertheless, these studies highlight the importance of the BMME in the regulation of normal hematopoiesis, and present an intriguing potential for novel pharmacological targets in leukemias.

## Leukemic Progression and Chemoresistance

The BMME becomes dramatically altered during leukemia; this has been demonstrated in multiple murine models of leukemia and is very well‐established in humans in hematological diseases such as multiple myeloma, a malignancy of plasma cells.^(^ ^69^ ^)^ Many of these changes, induced by the presence of malignant cells, overlap with the types of BMME dysfunction that can initiate leukemia; for example, abnormal cytokine signaling, MSC and osteolineage cell dysfunction, and vascular remodeling occur in MDS.^(^ ^70^, ^71^ ^)^ Such alterations support disease progression by providing a permissive environment for the replication of leukemic cells, as well as a haven for leukemic stem cells (LSCs) at the HSC niche during chemotherapy.

### LSCs can escape chemotherapy at an HSC niche

Cancer stem cells (CSCs), which can self‐renew and initiate a malignancy, were first discovered in leukemia,^(^ ^72^, ^73^ ^)^ and the concept is now applied to many solid tumors. LSCs, also called leukemia‐initiating cells (LICs), are the CSCs of leukemia; they can become quiescent at the HSC niche and escape chemotherapy, and these cells must be eradicated in order to clear the disease from being able to reconstitute to relapse.^(^ ^74^ ^)^ They replicate to produce more leukemic cells, although differentiation to normal hematopoietic lineages is blocked, producing an immature leukemic blast that may often express many markers of normal blood cells but does not act functionally as a fully differentiated hematopoietic cell.

Interaction with the BMME is capable of conferring chemotherapy resistance to malignant cells in the BM; leukemic cells that reside near the endosteum are selectively retained following treatment with chemotherapeutics.^(^ ^75^ ^)^ BM stromal cells have been well‐described to confer chemoresistance to AML cells, this has been primarily reported using in vitro co‐cultures.^(^ ^76^, ^77^, ^78^ ^)^ In in vivo models of both acute lymphoblastic leukemia (ALL) and AML, very late antigen‐4 (VLA‐4)/vascular cell adhesion protein 1 (VCAM‐1) signaling activated nuclear factor κB (NF‐κB) in MSCs and contributed to chemoresistance in LSC populations.^79^ ^)^ However, the precise population of cells and molecular mechanisms involved in chemoresistance have only recently begun to be elucidated.

Cancer‐associated fibroblasts (CAFs) have been found to support chemoresistance and, in the solid tumor microenvironment, they correlate with poor prognosis.^(^ ^80^ ^)^ This has also been found to occur in B cell acute lymphoblastic leukemia (B‐ALL), where CAFs are derived from MSCs^(^ ^81^ ^)^; these CAFs transfer mitochondria to B‐ALL cells to rescue them from chemotherapy‐induced ROS stress, and this process is reversible by using microtubule inhibitors. Furthermore, CAFs have been previously implicated to be supportive in the pathology of multiple myeloma.^(^ ^82^ ^)^ The concept that there are different subsets of fibroblasts, where some have an immunosuppressive phenotype while others do not support cancer progression, may be applicable to other cell types in the microenvironment. This is an expanding area of research, especially as the use of new methods, such as single‐cell RNA sequencing technology, emerges.

ECs have been shown to maintain chronic myelogenous leukemia (CML)‐initiating cells in a more quiescent state in a murine model, and targeting the adhesion molecule E‐selectin on vasculature may be a novel way to improve disease progression in myeloid leukemia.^(^ ^83^ ^)^ Inhibition of E‐selectin in combination with imatinib treatment has been shown to prolong survival of mice with CML.^(^ ^84^ ^)^ A similar phenomenon was also observed in AML, with E‐selectin inhibition coupled with chemotherapy, where leukemic mice doubled their survival time compared to animals receiving chemotherapy alone.^(^ ^37^ ^)^ Furthermore, ECs have been shown to produce large amounts of microRNA‐126 (miR‐126) in CML, and targeting of miR‐126 enhances the effectiveness of tyrosine kinase inhibitors, thereby reducing LSC populations in human CML xenografts.^(^ ^85^ ^)^ Additionally, the expression of CXCL12 by ECs has been shown to be important in the maintenance and chemoresistance of T cell acute lymphoblastic leukemia (T‐ALL).^(^ ^86^ ^)^ Thus, EC and MSC interactions with LSC populations highlight a therapeutic opportunity to improve response to traditional chemotherapy and reduce the rate of relapse.

### Inflammatory signals promote leukemic progression

In a model of CML, transgenic mice expressing the BCR/ABL (Philadelphia Chromosome) translocation in hematopoietic stem and progenitor cells (HSPCs) displayed BMME dysfunction that selectively supports LSCs over normal HSPCs.^(^ ^87^ ^)^ This altered support was attributable to the presence of high concentrations of the inflammatory chemokines CCL3/MIP‐1α and CCL4/MIP‐1β, as well as the cytokines IL‐1α, IL‐1β, and tumor necrosis factor α (TNF‐α), in the BM of leukemic mice.^(^ ^87^ ^)^ CCL3 was also produced by a basophil lineage cell and was critical for maintenance of an LSC population during CML.^(^ ^88^, ^89^ ^)^ CCL3 has been shown to mediate microenvironmental support for LSCs by causing a severe osteoblastic defect in a murine model of blast crisis CML (bcCML) that is suggested to be CCL3 dependent.^(^ ^29^ ^)^ Furthermore, it also alters differentiation of MSCs to produce an expanded and functionally deficient OB population during chronic‐phase CML.^(^ ^90^ ^)^ Loss of osteoblastic cells has previously been shown to result in accelerated leukemic progression in a CML model, suggesting that leukemia‐induced osteoblastic dysfunction contributes to microenvironmental support for leukemic cells.^(^ ^10^ ^)^ Finally, inhibition of CCL3 signaling in the BMME reduced leukemic burden in a murine bcCML model, when mice were treated with Maraviroc, a US Food and Drug Administration (FDA)‐approved CCR5 inhibitor (a receptor for CCL3), using a BM targeted nanoparticle delivery system.^(^ ^91^ ^)^

LSCs produce TNF‐α, and this upregulates the NF‐κB pathway in an autocrine manner, worsening disease progression in AML and CML.^(^ ^92^, ^93^ ^)^ Furthermore, leukemic cells can polarize macrophages to a leukemia‐supportive phenotype through growth factor independence 1 (Gfi1),^(^ ^94^ ^)^ a transcription factor necessary for the regulation of normal hematopoiesis that has been previously linked to leukemic development.^(^ ^95^, ^96^ ^)^

### Increased inflammation supports chemoresistance

When crosstalk between macrophages and MSCs is dysregulated in disease, it can promote retention of LSCs and escape from chemotherapy.^(^ ^97^ ^)^ It has been shown that a depletion of macrophages mobilizes HSCs,^(^ ^98^ ^)^ demonstrating their role in controlling HSC retention. Macrophages secrete inflammatory prostaglandin E2, signaling to *Nestin* ^+^ MSCs to secrete CXCL12, Ang‐1, and VCAM‐1,^(^ ^6^, ^14^, ^99^ ^)^ which are HSC/LSC retention factors and control HSC homing as shown in live tracking studies.^(^ ^100^ ^)^ Monocytes expressing alpha‐smooth muscle actin (α‐SMA^+^) also stimulate the same prostaglandin E2 signaling to *Nestin* ^+^ MSCs.^(^ ^101^ ^)^ Therefore, increased concentrations of CXCL12 in the BM may lessen the normal mobilization of HSCs, as well as leukemic cells, leading to retention in the BM and resistance to chemotherapy.^(^ ^97^ ^)^

Around one‐third of CD4^+^ T cells in the BM are regulatory T cells (Tregs) (CD4^+^, CD25^+^, FOXP3^+^), as compared to approximately one‐tenth of the cells present in the spleen.^(^ ^102^ ^)^ Tregs suppress myeloid differentiation of HSPCs, where they colocalize in the endosteum, and have been shown to regulate normal B cell lymphopoiesis.^(^ ^103^ ^)^ However, the presence of a higher percentage of Tregs in the BM can lead to an immune permissive environment where less immune cells will effectively attack the leukemic cells,^(^ ^16^ ^)^ and this may provide some resistance to immunotherapy.

## Microenvironmental Alterations Cause a Loss of Normal Hematopoiesis

Changes in the BMME occur with the development of hematological malignancies, leading to HSC niche dysfunction, and, thereby, result in the loss of normal hematopoiesis. Signals from leukemic cells lead to BMME remodeling, and the creation of a self‐reinforcing leukemic niche through positive feedback signaling loops. The loss of normal hematopoiesis may eventually cause pancytopenia, hemorrhage, and infections, leading to much of the morbidity and symptoms associated with leukemias. Therefore, research of the BMME in hematological malignancies is lending to the development of “nichotherapies”,^(^ ^104^ ^)^ in concert with chemotherapies, to improve prognosis by slowing disease progression and retaining support for normal hematopoiesis.

### The endosteal niche is remodeled by malignancy

In an immunocompetent mouse model of AML, OBs were significantly inhibited by a factor secreted by the leukemic cells, suggested to be the chemokine CCL3, that was also confirmed to be increased in the BM of primary patient samples of AML.^(^ ^29^ ^)^ Additionally, during a murine model of myeloproliferative neoplasia, MSCs differentiated into functionally‐altered osteoblastic lineage cells due to signals from leukemic myeloid cells that include TPO and CCL3, among other local paracrine signals, driving the expansion of the osteoblastic lineage cells. This creates an endosteal leukemic niche that supports inflammation and fibrosis, while losing important hematopoietic signals such as transforming growth factor β (TGF‐β) and other HSC retention factors,^(^ ^90^ ^)^ favoring LSCs over normal HSCs. Because the chemokine CCL3 has been demonstrated to be involved in BM microenvironmental dysfunction in multiple leukemic models and is not critical for the maintenance of non‐leukemic HSCs,^(^ ^30^ ^)^ it could be an intriguing therapeutic target.

To date, the nature of the cell types that form the endosteal niche is not clear. A recent study described that there are at least five different types of skeletal‐derived cell types that line endosteal surfaces, four of these co‐express CD51^+^, lack Sca‐1 expression, and can be further subdivided by their expression of platelet‐derived growth factor receptor α (PDGFRα) and PDGFRβ.^(^ ^22^ ^)^ These four novel skeletal cell types have been shown to have distinct functional potential, including support of B lymphopoiesis, and some of these cell types express high levels of HSC regulatory molecules. Multiplex immunofluorescence studies revealed that the four populations of CD51^+^ Sca‐1^−^ cells localized to the growth plate and/or trabecular, but not cortical, endosteal bone surfaces in tibias of mice. Furthermore, mature cuboidal osteoblasts lined trabecular and cortical bone and were shown to be CD51^−^.^(^ ^22^ ^)^ Hence, there are a hierarchy of cell types that line the endosteal surface and further studies of their roles in regulating hematopoiesis, including HSCs, and their responses to hematopoietic malignancies are warranted.

Leukemic exosomes secreting messenger RNAs (mRNAs) to the microenvironment in AML remodel the HSC niche.^(^ ^105^ ^)^ When mice were preconditioned with leukemic exosomes in the BM, the disease progressed more rapidly, and osteolineage cells had blunted development through the induction of Dickkopf Wnt signaling pathway inhibitor 1 (DKK1) overexpression. Additionally, factors that support the normal HSC niche, including CXCL12, were downregulated in stromal cells after exposure to AML‐derived exosomes, suppressing normal hematopoiesis and promoting a niche that is more permissive to malignant cell growth.

Intravital microscopy studies using a mouse model of T‐ALL revealed that the leukemic cells destroyed OBs, thereby altering the endosteal niche.^(^ ^106^ ^)^ A loss of OBs was also observed in sections of human T‐ALL BM trephine biopsies. Moreover, SNS signaling is disrupted due to development of sympathetic neuropathy in an mixed lineage leukemia (MLL)/AF9 model of AML,^(^ ^107^ ^)^ leading to niche dysfunction by reducing differentiation of *Nestin* ^+^ MSCs, a key perivascular cell signal for HSC self‐renewal and maintenance. Such SNS signaling is also perturbed in myeloproliferative neoplasms (MPNs) in humans, and the ablation of *Nestin* ^+^ MSCs caused an expansion of myeloproliferative progenitor populations in a mouse model of MPN.^(^ ^108^ ^)^

The endosteal vascular niche is remodeled in a murine model of AML by leukemia cell‐secreted anti‐angiogenic and inflammatory cytokines including TNF and CXCL2.^(^ ^38^ ^)^ Together, these impair the ability to sustain normal hematopoiesis as the endosteal niche loses cells that support normal HSC maintenance, due to damage to stromal cells, ECs, and OBs. The use of therapeutic deferoxamine or genetic targeting to protect the endosteal vasculature was able to rescue some HSC loss and slow disease progression, highlighting the importance of endosteal vasculature to contribute to a normal functioning endosteal HSC niche. Additionally, leukemic precursor cells caused remodeling of the niche by CLXL12 reduction, leading to a growth advantage to ALL cells over normal hematopoietic cells in a human ex vivo co‐culture study.^(^ ^109^ ^)^ Finally, a common complication after therapies such as chemotherapy or radiation is the bone loss secondary to inflammation, altering the production of myeloid lineage cells.^(^ ^41^, ^110^ ^)^

### Chronic inflammation contributes aberrant signals

During long‐term stress response, caused by a lack of mature blood cells during leukemia, and/or by the loss of blood cells after chemotherapy, HSPCs are prompted to increase blood cell production by responding to incoming inflammatory signals and cytokines, such as IFNs.^(^ ^111^ ^)^ This stimulates “emergency” hematopoiesis, especially for short‐lived cells such as granulocytes that cannot undergo clonal expansion.^(^ ^112^ ^)^ In mouse models of BMME‐induced blood cell malignancies an inflammatory phenotype has also been observed, such as in the *RAR‐γ* and *Rb* deficient mice.^(^ ^39^, ^41^ ^)^ However, chronic signaling for such hematopoiesis can lead to HSC exhaustion, and/or a loss of the HSC pool, where HSCs are no longer able to self‐renew to produce the primary differentiated cells that are the progenitors to the lymphoid and myeloid lineages.^(^ ^113^ ^)^ Therefore, inflammatory signals from immune cells may contribute to a loss of hematopoiesis when these signals are prolonged in the BMME during leukemia.

Chronic proinflammatory responses can increase the number of active HSCs that have decreased functionality.^(^ ^114^ ^)^ During disease, antigens on leukemic cells can activate T cells to become effector T cells (CD8^+^), and it has been shown that some T cells may be activated to attack leukemic cells,^(^ ^115^ ^)^ though this is dampened in an immune permissive environment when high levels of Tregs are present during leukemias.^(^ ^116^ ^)^ Cytotoxic effector CD8^+^ T cells (CTLs) also secrete the proinflammatory cytokine IFN‐γ, which can directly and/or indirectly stimulate HSPCs to proliferate; for example, IFN‐γ activates MSCs to secrete IL‐6, which in turn has been shown to induce proliferation of LSCs in a murine CML model.^(^ ^117^ ^)^ Immune cells also produce IL‐6, such as T cells and B cells, and IL‐6 signaling has been shown to progress disease in a CML model through a paracrine proinflammatory positive feedback loop.^(^ ^118^ ^)^

The inflammatory cytokine IFN‐γ is secreted by activated T cells and macrophages during normal immune response, and it can also stimulate quiescent HSCs to actively replicate.^(^ ^119^ ^)^ Furthermore, elevated IFN‐γ has negative impacts on the stemness and engrafting capabilities of HSCs, while increasing proliferation of HSPCs.^(^ ^120^ ^)^ The signaling from IFN‐γ stimulates the expression of BST2, an E‐selectin ligand, on HSCs, leading them to move away from the CAR cells that are vital to retain their quiescence.^(^ ^121^ ^)^ Additionally, HSPCs with mutant DNMT3a are particularly responsive to IFN‐γ signaling, a mechanism that allows them to drive premalignant clonal hematopoiesis.^(^ ^122^ ^)^ Both T cells and B cells also secrete TNF‐α, a major proinflammatory cytokine, which, like IFN‐γ, has been shown to repress functional hematopoiesis,^(^ ^123^ ^)^ and these cell types are persistent during chronic inflammation. Both TNF‐α and IFN‐γ have been shown to be elevated in patients with MDS.^(^ ^124^ ^)^

### The HSC niche microenvironment is affected across leukemia types

These studies support the concept that the recovery of normal hematopoiesis from leukemia‐induced impairment requires not only elimination of leukemic cells, but also restoration of the normal BMME; combination therapies that include targeting the microenvironment will add benefit over traditional chemotherapy to leukemic cells. Additionally, although a large variety of cell populations are involved in BM dysfunction during leukemia, there are consistent features that emerge, namely the involvement of MSCs, osteolineage cells, and ECs, and inflammatory signaling, suggesting that therapeutic targets may exist that would be effective across multiple leukemic subtypes.

## Conclusions

The studies reviewed herein, largely performed in mouse models, reveal that leukemias of both myeloid and lymphoid origin reside in the BM and interact extensively with the BMME. The cellular populations and several molecular mechanisms involved in leukemic progression also often overlap, with mesenchymal lineage cells and endothelial cells being common targets of leukemia‐derived inflammatory signals (CCL3, TNF, CXCL12) identified across multiple leukemic subtypes. Therefore, continued investigation into these possible novel therapeutic targets that have the potential to affect multiple subtypes of leukemia is critical.

Hindering these research efforts is the lack of a reliable in vitro model of the human leukemic BMME, although there are models that have moved the field closer to being able to mimic the human leukemic BMME. One such model used subcutaneous humanized ossicles to engraft human HSPCs and primary AML samples in mice with a more relevant human BMME.^(^ ^125^ ^)^ This model and the development of others will be useful in the validation of findings generated in mouse models, with a direction to translational human studies.

To note, BMME‐induced hematological malignancies, in murine research, have resulted primarily in MPS‐like disease that in some cases can progress to fully transformed acute leukemia, and do not typically result in a de novo AML/ALL. Therefore, we propose that some hematopoietic malignancies may be able to be induced extrinsically, and in others, the BMME alterations are in addition to pathogenesis from cell‐intrinsic mechanisms leading to disease development. Here, the leukemia develops and then benefits from BMME dysfunction. Regardless, what has become clear is that virtually every hematopoietic malignancy involves the BMME in some fashion, including: initiation, progression, resistance to chemotherapy, and suppression of normal hematopoiesis, making the leukemic BMME a prime target for future research.

## Disclosures

Celia A. Soto, Cristina Lo Celso, Louise E. Purton, and Benjamin J. Frisch have no real or potential conflicts of interest to declare.

## Author contributions

Celia A. Soto, Cristina Lo Celso and Louise E. Purton wrote, reviewed and edited the manuscript. Benjamin J. Frisch wrote, reviewed and edited the manuscript while supervising all aspects of manuscript preparation.

### Peer review

The peer review history for this article is available at https://publons.com/publon/10.1002/jbm4.10516.
